# G9A promotes tumor cell growth and invasion by silencing CASP1 in non-small-cell lung cancer cells

**DOI:** 10.1038/cddis.2017.65

**Published:** 2017-04-06

**Authors:** Tianhao Huang, Peng Zhang, Wang Li, Tian Zhao, Zhixiong Zhang, Sujun Chen, Yan Yang, Yonghong Feng, Fei Li, X Shirley Liu, Lei Zhang, Gening Jiang, Fan Zhang

**Affiliations:** 1 Shanghai Key Lab of Tuberculosis, Shanghai Pulmonary Hospital, Tongji University School of Medicine, 507 Zhengmin Road, Shanghai 200433, China; 2 Clinical Translational Research Center, Shanghai Pulmonary Hospital, Tongji University School of Medicine, 507 Zhengmin Road, Shanghai 200433, China; 3 School of Life Science and Technology, Tongji University, 1239 Siping Road, Shanghai 200092, China; 4 Department of Thoracic Surgery, Shanghai Pulmonary Hospital, Tongji University School of Medicine, 507 Zhengmin Road, Shanghai 200433, China; 5 Department of Biology, New York University, New York, NY 10003, USA; 6 Department of Biostatistics and Computational Biology, Dana-Farber Cancer Institute and Harvard School of Public Health, Boston, MA 02215, USA

## Abstract

Non-small-cell lung cancer (NSCLC) is one of the leading causes of cancer-related death worldwide. Although epigenetic deregulation is known to be important for tumor progression, the molecular mechanisms in NSCLC remain unclear. Here, we found that G9A (known as EHMT2), a histone methyltransferase responsible for mono- or di-methylation of histone 3 (H3) lysine 9 (K9), is significantly upregulated in NSCLC. Knocking down G9A or pharmacological inhibition of its activity suppressed tumor cell growth, colony formation, invasion and migration. Furthermore, G9A exerts these functions by repressing CASP1 expression. Knocking down CASP1 in G9A-deficient cell restored capacities of tumor cell invasion and migration. Mechanistically, G9A silences the CASP1 promoter activity by increasing H3K9me2 around its promoter. Finally, high expression of G9A or low expression of CASP1 is correlated with poor overall survival in lung adenocarcinoma. Overall, our study uncovers a novel mechanism of G9A promoting tumor cell growth and invasion by silencing CASP1, and implies that G9A may serve as a therapeutic target in treating NSCLC.

Lung cancer is a leading cause of death in all types of cancers. Non-small-cell lung cancer (NSCLC) is the major type of lung cancer. It is a heterogeneous disease; many different oncogenic mutations have been identified. Epigenetic deregulation is implicated in tumor development.^1^ Histone methylation is one of primary epigenetic modifications affecting gene expression, and is involved in many cellular processes.^2^

G9A/EHMT2 is a histone lysine methyltransferase that specifically mono- and dimethylates Lys9 of histone H3 (H3K9me1 and H3K9me2, respectively).^3, 4, 5^ It is overexpressed in many types of cancer,^6, 7, 8, 9, 10^ and its higher expression is associated with poor survival of cancer patients.^6, 9, 11^ Mechanistically, G9A acts as a transcriptional repressor to silence gene expression.^12, 13^ For example, G9A interacts with Snail, a transcriptional factor, and is critical for Snail-mediated E-cadherin repression in human breast cancer.^14^ Moreover, hypoxic stress induced accumulation of G9A leads to increased H3K9me2 and repression of its target genes to promote cell survival.^15^ However, G9A also functions as a transcriptional activator depending on its interacting cofactors.^16^ For example, G9A can epigenetically activate the serine–glycine synthesis pathway to sustain cancer cell survival and proliferation.^17^ However, its role in NSCLC is not well understood. Identification of its key target genes or pathways will help to understand the molecular mechanism of tumorigenesis and metastasis in NSCLC.

CASP1, also known as caspase 1, belongs to the family of CASP proteins, which are cysteine proteases regulating many cellular processes, such as apoptosis, inflammation and necrosis, etc.^18, 19^ Specifically, CASP1 mediated inflammasome activation regulated immune response and disease pathogenesis.^20^ In addition, CASP1-induced pyroptosis is an innate immune effector mechanism against intracellular bacteria.^21, 22^ However, the function and regulation of CASP1 in NSCLC is poorly understood.

In this study, we examined the biological function of G9A in NSCLC cells, and identified one of its key target genes, CASP1. We also uncovered the molecular mechanism of how G9A represses CASP1 to promote tumor cell growth and invasion. Finally, we analyzed whether G9A or CASP1 could serve as prognostic biomarkers in lung adenocarcinoma (LUAD). In addition, our study suggests that G9A may be a therapeutic target for NSCLC.

## Results

### G9A expression is aberrantly elevated in NSCLC patients

**Figure 1:**
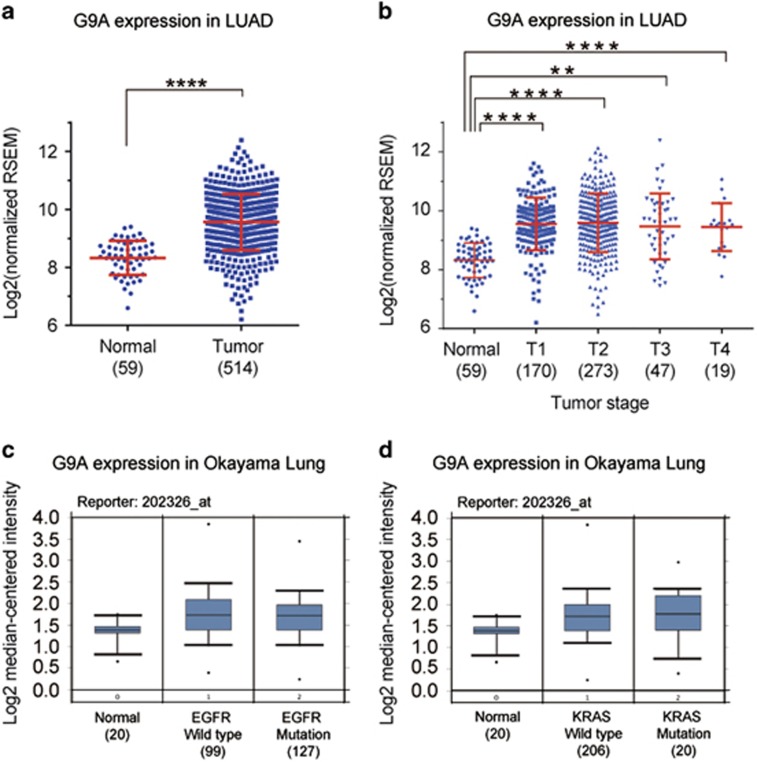
G9A is aberrantly upregulated in NSCLC. (**a**) Relative expression of G9A in the normal and tumor samples of LUAD (lung adenocarcinoma) from the TCGA database. The log2 fold change and *P*-value of G9A expression is 0.76 (*P*=4.31E-06, Cancer *versus* Normal). The number in the parenthesis represents the sample size. (**b**) Relative expression of G9A in the normal and different T stages of tumor samples of LUAD from the TCGA database. Log2 fold changes and *P*-values of G9A expression are 0.73 (*P*=7.41E-06, T1 *versus* Normal), 0.80 (*P*=2.56E-6, T2 *versus* Normal), 0.82 (*P*=1.12E-05, T3 *versus* Normal) and 0.72 (*P*=0.0082, T4 *versus* Normal). (**c**) Relative expression of G9A in the normal lung tissues and LUAD patients with either a wild type or mutant EGFR gene in the Okayama Lung data set (from the Oncomine database). (**d**) Relative expression of G9A in the normal lung tissues and LUAD patients with either a wild type or mutant KRAS gene in the Okayama Lung data set. Reporter stands for the probe name used in the experiments. The number in the parenthesis represents the sample size

To examine whether G9A expression is dysregulated in NSCLC, we compared its expression between normal and cancer samples using the mRNA-Seq data of LUAD from the TCGA database. We found that G9A is significantly upregulated in tumor samples compared with the normal control in LUAD (Figure 1a). In addition, G9A is upregulated in all stages of LUAD compared with the normal control (Figure 1b).

We also examined the expression of G9A in lung cancer using the oncomine database, and found that G9A is upregulated in LUAD regardless of EGFR or KRAS mutation status (Figures 1c and d). Overall, this analysis indicates that G9A is abnormally elevated in LUAD of NSCLC compared with the normal lung tissues.

### G9A promotes tumor cell growth and invasion in NSCLC

**Figure 2:**
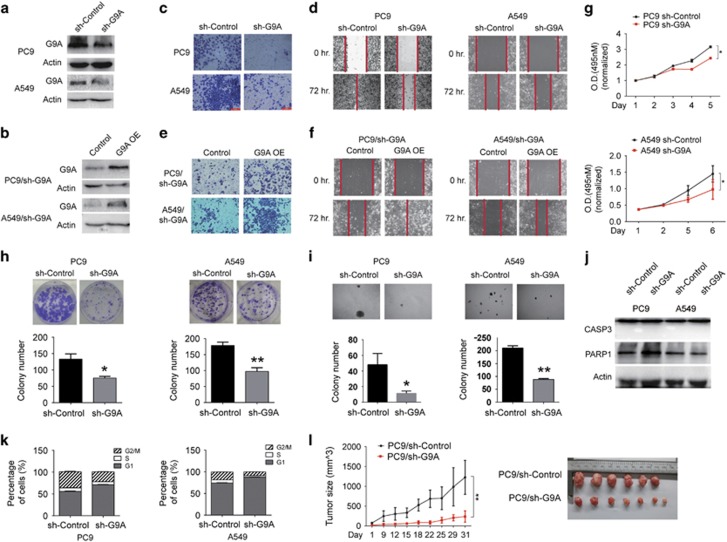
G9A promotes tumor growth and metastasis in NSCLC. (**a**) G9A knockdown was detected by WB in PC9 or A549 cells stably expressing the control shRNA (sh-Control) or G9A shRNA (sh-G9A). (**b**) G9A overexpression was detected by WB in G9A-depleted PC9 or A549 cells transfected with the control plasmid (Control) or G9A overexpression plasmid (G9A OE). Actin serves as the loading control. (**c** and **d**) Cell invasion (**c**) and migration (**d**) assays were carried out in PC9 or A549 cells stably expressing the control shRNA or G9A shRNA. The red line indicates the edge of migrating cells at a given time point. (**e** and **f**) Cell invasion (**e**) and migration (**f**) assays were carried out in G9A-depleted PC9 or A549 cells transfected with the control plasmid or G9A overexpression plasmid. (**g**) Cell proliferation assays were carried out in PC9 (upper panel) or A549 (lower panel) cells stably expressing the control shRNA or G9A shRNA. (**h** and **i**) Colony formation assays (**h**) and sphere formation assays (**i**) in soft agar were carried out in PC9 (left panel) or A549 (right panel) cells stably expressing the control shRNA or G9A shRNA. Bar graphs show the quantification of average colony number in each condition. Each condition was performed in three replicates. (**j**) WB detection of CASP3 and PARP1 in PC9 or A549 cells stably expressing the control shRNA or G9A shRNA. Actin serves as the loading control. (**k**) Cell cycle assays were carried out in PC9 (left panel) or A549 (right panel) cells stably expressing the control shRNA or G9A shRNA. (**l**) Xenograft assays were carried out in nude mice using PC9 cells stably expressing the control shRNA or G9A shRNA. Tumor size was measured every 3 or 4 days for 5 weeks (left panel). In the end, xenograft tumors were dissected and photographed (right panel). Each group contained six or seven mice

To investigate the function of G9A in NSCLC cells, we knocked down the level of G9A protein significantly in PC9 and A549 cells by selecting cells stably expressing G9A shRNA (Figure 2a), and found that cell invasion and migration capacities were reduced considerably in these cells (Figures 2c and d).

Conversely, when overexpressing G9A in PC9 and A549 cells stably expressing G9A shRNA (Figure 2b), we observed that cell invasion and migration capacities were enhanced significantly in these cells (Figures 2e and f). In addition, these cells also had a slower cell proliferation rate (Figure 2g). Transient depletion of G9A by siRNA transfection in these cells also showed the similar phenotypes as cells with stable knockdown of G9A (Supplementary Figures S1A–E).

Similarly, colony formation and sphere formation abilities were reduced upon stable G9A knockdown in both PC9 and A549 cells (Figures 2h and i). Furthermore, cell cycle progression was delayed due to the prolonged G1 phase and shortened the G2 and M phases (Figure 2k), but the apoptosis rate was not significantly affected in cells with stable G9A knockdown, demonstrated by the lack of cleavage products of CASP3 and PARP1 proteins (two markers for apoptosis) in WB assays (Figure 2j).

To study whether G9A knockdown affects tumor cell growth *in vivo*, we carried out xenograft assays in nude mice, and found that tumors derived from cells stably expressing G9A shRNA showed significantly slower growth rates and smaller tumor sizes, compared with those derived from cells expressing the control shRNA (Figure 2l). We also carried out immunohistochemistry analysis, and found that the intensity for cell proliferation marker (Ki67) was considerably reduced in xenograft tumor depleted with G9A (Supplementary Figure S2A), which indicates that G9A knockdown reduced cell proliferation *in vivo*. Altogether, we conclude that G9A promotes tumor cell growth and invasion in NSCLC cells.

### CASP1 expression is repressed by G9A

**Figure 3:**
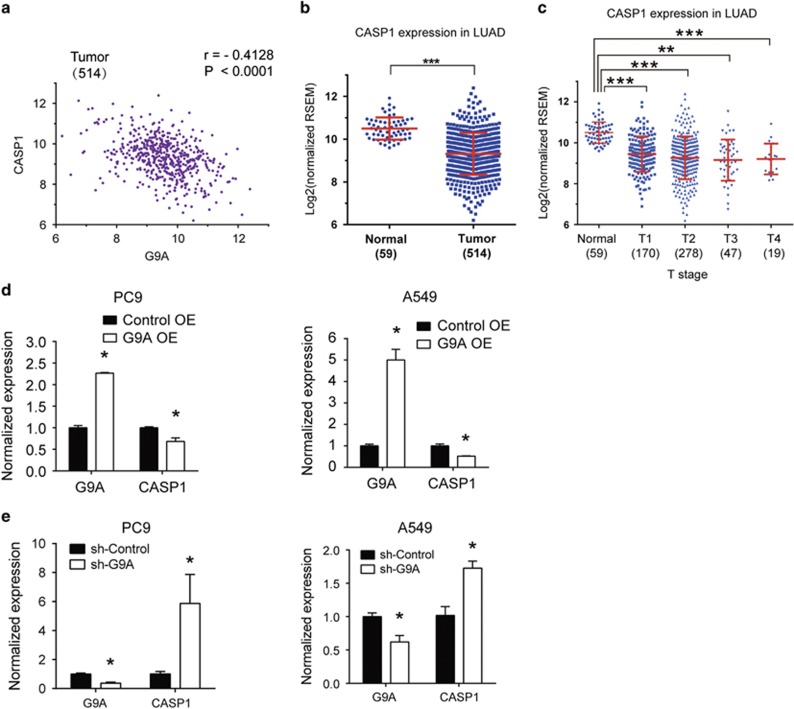
CASP1 expression is negatively correlated with G9A, and is repressed by G9A in NSCLC. (**a**) Gene expression correlation analysis between G9A and CASP1 in tumor samples of LUAD. Correlation coefficient (*r*)=−0.4128, *P*-value (P)<0.0001. The number in the parenthesis represents the sample size. (**b**) Relative expression of CASP1 in the normal and tumor tissues in LUAD. The log2 fold change and *P*-value of CASP1 expression are: −0.97 (*P*=5.67E-10, Cancer *versus* Normal). (**c**) Relative expression of CASP1 in different stages (T1, T2, T3 and T4) of tumor tissues of LUAD. Log2 fold changes in CASP1 expression are: −0.89 (*P*=1.52E-08, T1 *versus* Normal), −0.97 (*P*=9.48E-10, T2 *versus* Normal), −1.06 (*P*=1.24E-07, T3 *versus* Normal) and −1.11 (*P*=0.00085, T4 *versus* Normal). (**d**) RT-qPCR detection of G9A and CASP1 expression in PC9 or A549 cells transiently transfected with the control plasmid (Control OE) or G9A overexpression plasmid (G9A OE). (**e**) RT-qPCR detection of G9A and CASP1 expression in PC9 or A549 cells stably expressing the control shRNA (sh-Control) or G9A shRNA (sh-G9A). For all RT-qPCR, each condition was performed in triplicates. Data are represented as mean (S.D.)

To study the molecular mechanism of G9A-mediated tumor growth and invasion, first, we aimed to identify target genes of G9A in NSCLC cells, by carrying out gene expression correlation analysis in LUAD using the data downloaded from the cbioPortal website, and found that CASP1 expression is negatively correlated with G9A expression in NSCLC (Figure 3a). In addition, the expression of CASP1 is significantly downregulated in every T stage in LUAD compared with the normal tissue control (Figure 3b). Furthermore, we examined the correlation of G9A and CASP1 expression in different stages of LUAD, and found that they are also significantly negatively correlated in T1 and T2 stages (Figure 3c).

To study whether G9A represses CASP1 expression in NSCLC cells, we overexpressed G9A in PC9 and A549 cells, and found that CASP1 expression was repressed (Figure 3d). Conversely, depletion of G9A by shRNA reactivated CASP1 expression in these cells, validated by RT-qPCR (Figure 3e), indicating G9A represses CASP1 expression in these cells.

Therefore, our results indicate that G9A suppresses CASP1 gene expression in NSCLC cells, and their expressions are significantly negatively correlated between each other.

### CASP1 suppresses G9A-mediated cell invasion and migration

**Figure 4:**
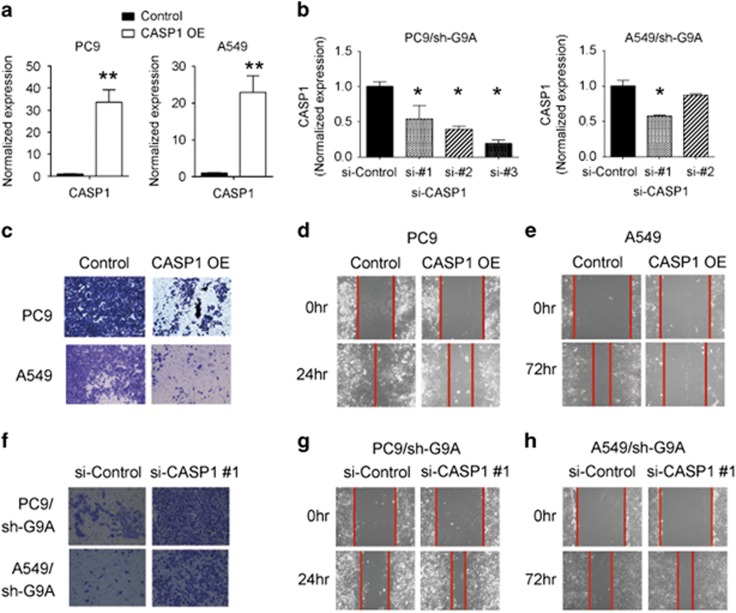
CASP1 suppresses G9A-mediated cell invasion and migration. (**a**) RT-qPCR detection of CASP1 expression in PC9 and A549 cells transfected with the control plasmid (Control) or CASP1 overexpression plasmid (CASP1 OE). (**b**) RT-qPCR detection of CASP1 expression in PC9 and A549 cells transfected with the control siRNA (si-Control) or three different CASP1 siRNAs (si-CASP1 #1, #2, and #3). (**c**) Cell invasion assays using PC9 or A549 cells transfected with the control plasmid (Control) or CASP1 overexpression plasmid (CASP1 OE). (**d** and **e**) Cell migration assays using PC9 or A549 cells transiently transfected with the control plasmid or CASP1 overexpression plasmid. The red line indicates the edge of migrating cells at a given time point. (**f**) Cell invasion assays using PC9 or A549 cells transiently transfected with the control siRNA (si-Control) or CASP1 siRNA (si-CASP1 #1). (**g** and **h**) Cell migration assays using PC9 or A549 cells transiently transfected with the control siRNA or CASP1 siRNA

To investigate whether repression of CASP1 expression is necessary for G9A-mediated cell invasion and migration, we transiently overexpressed CASP1 in PC9 and A549 cells (Figure 4a), and found that CASP1 overexpression significantly inhibited tumor cell invasion and migration abilities in these cells (Figures 4c–e). By contrast, when CASP1 was knocked down by siRNA in G9A-depleted tumor cells (Figure 4b), the invasion and migration capabilities of these cells were rescued (Figures 4f–h).

To investigate the biological function of CASP1 in tumorigenesis, we carried out bioinformatics analysis to examine the cellular pathways either positively or negatively correlated with the CASP1 expression in four different cancer types, including LUAD, breast cancer (BRCA), colon adenocarcinoma (COAD) and lung squamous cell carcinoma (LUSC) from the TCGA database (Supplementary Figure S3). We found that CASP1 expression is positively associated with pathways including cytokine–cytokine receptor interaction in LUAD, BRCA and LUSC, and metabolic pathways in COAD, but negatively associated with pathways, such as tight junction in LUAD, endocytosis, AMPK and mTOR signaling in BRCA and COAD (Supplementary Figure S3).

In conclusion, our results indicate that CASP1 overexpression suppresses G9A-mediated cell invasion and migration. The expression of CASP1 is either positively or negatively associated with distinctive pathways in cancer.

### G9A knockdown reduces the level of H3K9me2 at the CASP1 promoter and its overexpression represses the CASP1 promoter activity

G9A is a histone methyltransferase that increases the level of H3K9me2 around the promoter region of its target genes, and interacts with other co-repressors to silence target gene expression.^5, 12, 23, 24^

**Figure 5:**
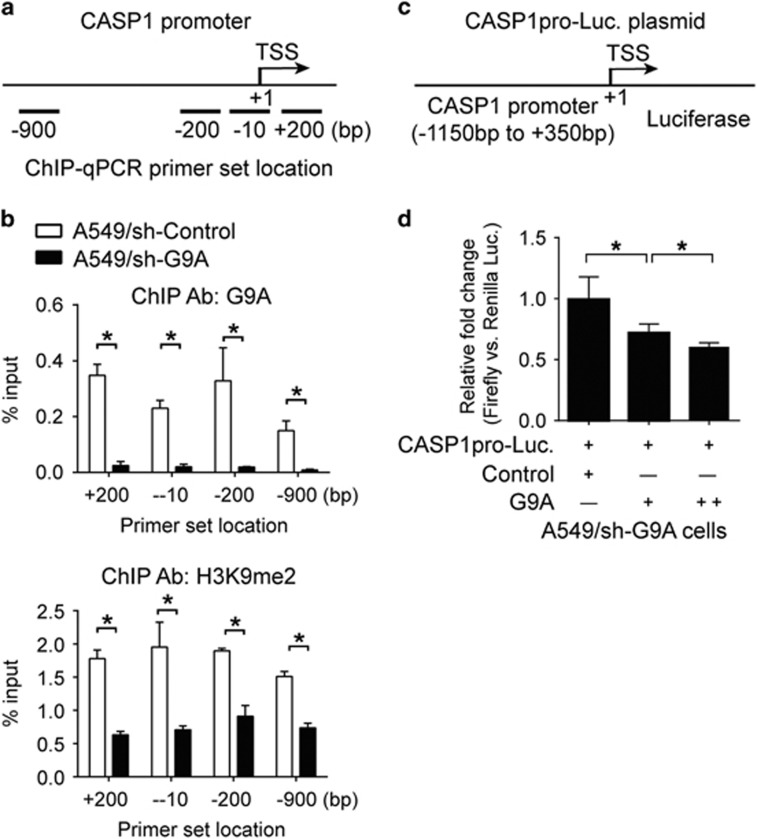
G9A knockdown reduces the level of H3K9me2 at the CASP1 promoter and G9A overexpression represses the CASP1 promoter activity. (**a**) Schematic drawing of the CASP1 promoter region. The positions of ChIP-qPCR primer set were labeled relative to the transcriptional start site (TSS). TSS is assigned as the '+1' position. (**b**) ChIP-qPCR was performed in A549 cells stably expressing the control shRNA (sh-Control) or G9A shRNA (sh-G9A) using anti-G9A (upper panel) or anti-H3K9me2 (lower panel) antibodies. The bar graph shows percentages of relative fold enrichment of G9A (upper panel) or H3K9me2 (lower panel) at different positions across the CASP1 promoter region, compared with the input. (**c**) Schematic drawing of the CASP1 promoter luciferase reporter construct (CASP1pro-Luc plasmid). The proximal promoter region (−1150 to +350 bp) of the CASP1 gene were cloned into the luciferase reporter vector (pGL3). (**d**) Luciferase assays were carried out in G9A-depleted A549 cells transfected with CASP1pro-Luc plasmid, and various amounts of the control plasmid (Control) or G9A overexpression (G9A) plasmid. Relative (Firefly *versus* Renilla) luciferase activities were determined. Each condition was performed in six replicates. Data are represented as mean (S.D.)

To investigate whether depletion of G9A reduced the level of H3K9me2 around the CASP1 gene promoter, we carried out chromatin immunoprecipitation assay using anti-G9A and anti-H3K9me2 antibodies, followed by qPCR to examine the relative enrichment of H3K9me2 around the promoter region of CASP1 gene, compared with the input (Figure 5a). We found that, in A549 cells stably expressing G9A shRNA, the levels of G9A were greatly reduced at the promoter region of CASP1 compared with those in cells expressing the control shRNA (Figure 5b, upper panel). Similarly, the levels of H3K9me2 were significantly reduced at the same regions around the CASP1 promoter in these cells as well (Figure 5b, lower panel).

To study whether G9A represses the CASP1 promoter activity, we cloned the CASP1 gene promoter into pGL3 vector, carried out luciferase reporter assays and found that G9A overexpression significantly suppressed the promoter activity of CASP1 promoter, and this effect was dosage dependent (Figures 5c and d). Overall, our results reveal that G9A silences the CASP1 promoter activity by inducing H3K9me2 around its promoter.

### Pharmacological inhibition of G9A inhibits cell proliferation, invasion and migration in NSCLC cells

To study whether pharmacological inhibition of G9A activity can inhibit tumor cell growth, invasion and migration, we treated PC9 and A549 cells with various concentrations (1, 5, 10 *μ*M) of BIX-01294, which is a small molecular inhibitor of G9A widely used in other cancer studies.^25^

**Figure 6:**
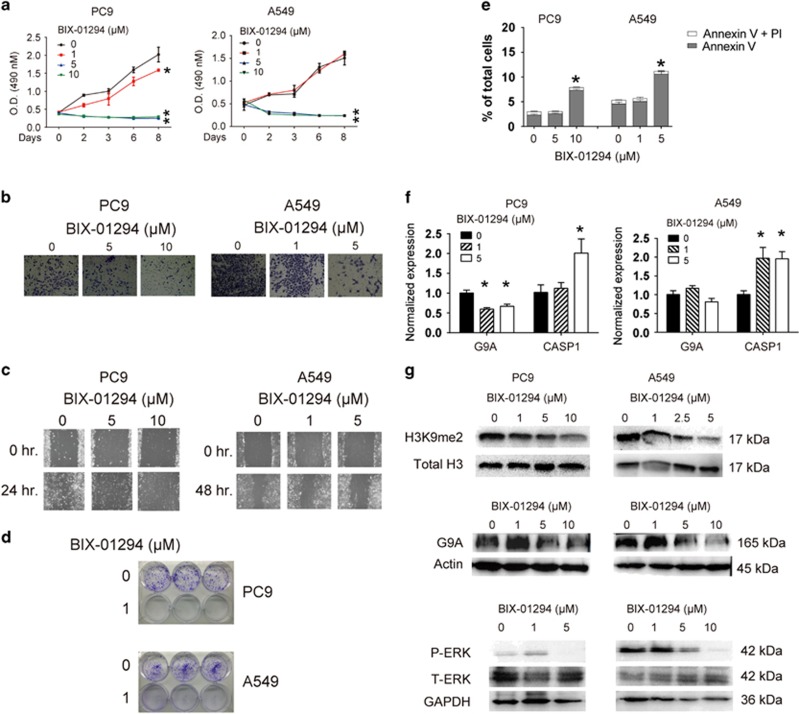
BIX-01294 treatment suppresses cell proliferation, invasion and migration in NSCLC cells. (**a**) Cell proliferation assays were carried out in PC9 and A549 cells treated with 0, 1, 5 or 10 *μ*M BIX-01294, at different time points. (**b**) Cell invasion assays were carried out in PC9 and A549 cells treated with 0, 1 and 5 *μ*M BIX-01294. (**c**) Cell migration assays were carried out in PC9 and A549 cells treated with 0, 1 and 5 *μ*M BIX-01294. The red line indicates the edge of migrating cells at a given time point. (**d**) Colony formation assays were carried out in PC9 and A549 cells treated with 0 and 1 *μ*M BIX-01294. (**e**) Apoptosis assays using the BD Annexin V FITC kit were carried out in PC9 and A549 cells treated with 0, 1 and 5 *μ*M BIX-01294. The bar graph represents percentages of cells expressing early (Annexin V) or late (PI) apoptosis markers. (**f**) RT-qPCR detection of CASP1 and G9A expression in PC9 and A549 cells treated with 0, 0.5, 1 and 5 *μ*M BIX-01294. Each condition was performed in triplicates. Data are represented as mean (S.D.). (**g**) Upper and middle panels: WB detection of H3K9me2 and G9A in PC9 and A549 cells treated with 0, 1, 2.5, 5 or 10 *μ*M BIX-01294. The total level of histone H3 and actin serve as loading controls. Lower panel: WB detection of the total (T-) level of and phosphorylated (P-) ERK kinase in PC9 and A549 cells treated with 0, 1, 5 or 10 *μ*M BIX-01294. GAPDH serves as the loading control

We found that 1 or 5 *μ*M BIX-01294 treatment significantly inhibited the proliferation rate of PC9 or A549 cells, respectively (Figure 6a), and 5 *μ*M BIX-01294 suppressed cell invasion and migration in both cells (Figures 6b and c). In addition, as low as 1 *μ*M BIX-01294 treatment significantly inhibited colony formation of these cells (Figure 6d).

To study whether these changes were due to enhanced apoptosis induced by BIX-01294, we treated these cells with 1, 5 or 10 *μ*M of the inhibitor, and found that, 5  or 10 *μ*M BIX-01294 treatment caused significantly increased apoptosis rate in A549 or PC9 cells, respectively, compared with the control treatment (Figure 6e). It seems that A549 cells are more sensitive to BIX-01294-mediated apoptosis than PC9 cells.

To determine whether inhibiting the enzymatic activity of G9A can reactivate CASP1 expression, we treated these cells with 0, 1 and 5 *μ*M BIX-01294, and found that 5 *μ*M BIX-01294 in PC9 cells, but as low as 1 *μ*M BIX-01294 in A549 cells significantly reactivated CASP1 expression (Figure 6f).

Interestingly, we found that as low as 1 *μ*M BIX-01294 treatment could reduce the total level of H3K9me2 in both PC9 and A549 cells, and the level of G9A was decreased at 5 *μ*M BIX-01294 treatment (Figure 6g). To assess the effect of this inhibitor on the signaling pathway, we treated these cells with 1, 5 and 10 *μ*M BIX-01294, and found that 5 *μ*M BIX-01294 significantly suppressed phosphorylation of the ERK kinase in both cells (Figure 6g).

Taken together, these results indicate that inhibition of G9A enzymatic activity can suppress G9A-mediated tumor cell growth, invasion and migration, which is consistent with our data using G9A-depleted NSCLC cells.

### High expression of G9A and low expression of CASP1 are significantly associated with poor overall survival in NSCLC patients

**Figure 7:**
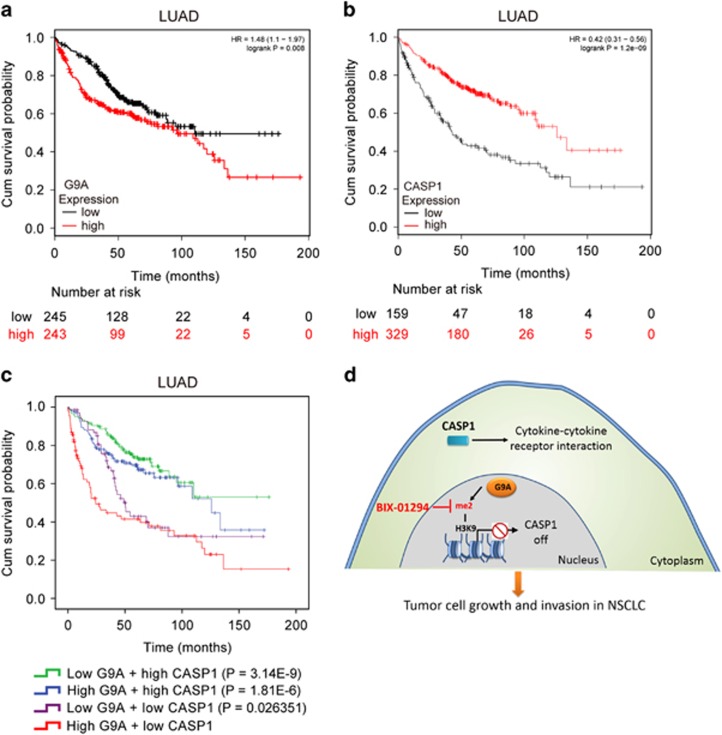
Kaplan–Meier curve analysis of overall survival probabilities of patients with lung adenocarcinoma based on G9A and CASP1 expression. (**a**) Kaplan–Merier survival analysis of LUAD patients based on G9A expression. Red line: high G9A expression; black line: low G9A expression. Numbers at risk for each time point were listed below time points. (**b**) Kaplan–Merier survival analysis of LUAD patients based on CASP1 expression. Red line: high CASP1 expression; black line: low CASP1 expression. (**c**) Kaplan–Merier survival analysis of LUAD patients based on both G9A and CASP1 expression. LUAD patients were divided into four groups: low G9A and high CASP1 expression (green line), high CASP1 and CASP1 expression (blue line), low G9A and CASP1 expression (purple line), and high G9A and low CASP1 expression (red line). *P*-values (from the log-rank test) were calculated using the group of high G9A and low CASP1 expression as the reference group. (**d**) A model for G9A-mediated tumor cell growth and invasion in NSCLC cells

To investigate whether the expression of G9A or CASP1 can serve as prognostic markers for NSCLC, we downloaded the clinical data of 488 patients with LUAD from a public database (http://kmplot.com/),^26^ and carried out Kaplan–Meier survival analysis. We found that high expression of G9A is associated with poor overall survival in LUAD (Figure 7a), while high expression of CASP1 is associated with better overall survival in LUAD (Figure 7b), with log-rank (Mantel–Cox) *P*-values of 0.008 for G9A and 1.2e-09 for CASP1, respectively.

To further study the effect of the expressions of both genes on patient overall survival, we divided the LUAD patients into four groups: high or low expressions of both G9A and CASP1, low expression of G9A and high expression of CASP1, and high expression of G9A and low expression of CASP1. Using the log-rank test, we found that the most significant difference in patient survival is between the group carrying high expression of G9A and low expression of CASP1 and the group carrying low expression of G9A and high expression of CASP1, with log-rank *P*-value of 3.14E-09 (Figure 7c and Supplementary Table S1).

Therefore, we conclude that high expression of G9A or low expression of CASP1 may be used as a poor prognostic marker for LUAD patients.

## Discussion

In this study, we found that histone methyltransferase G9A is aberrantly upregulated in NSCLC, and it promotes cancer cell growth, colony formation, invasion and migration, as well as enhances tumor growth *in vivo*. In addition, G9A mediates these effects through silencing expression of CASP1. Pharmacological inhibition of G9A has the strong antitumor effect in NSCLC cells. Finally, high expression of G9A and low expression of CASP1 indicate poor prognosis in LUAD patients.

Based on these findings, we proposed a mechanistic model here (Figure 7d): G9A silences CASP1 expression by increasing the level of H3K9me2 around its promoter region. Decreased CASP1 expression is associated with changes in cytokine–cytokine receptor interaction and tight junction pathway possibly, which may lead to enhanced cell proliferation, migration and invasion in NSCLC cells.

It is possible that G9A regulates other genes and pathways in NSCLC; for example, G9A was found to mediate tumor metastasis by epigenetically repressing the cell adhesion molecule EPCAM in lung cancer cells.^27^ In addition, G9A can suppress the transcription of the SIAH1 gene, which encodes a member of E3 ubiquitin ligases and functions as a tumor suppressor, in lung cancer cells.^6^ Here, we demonstrates that CASP1 is one of the important target genes of G9A in NSCLC. How G9A is recruited to the promoter of CASP1 remains to be determined, it may require additional factors. For example, G9A is recruited by LSH, a member of SNF2 family of ATP-dependent chromatin enzymes, to induce DNA methylation and stable gene silencing during development.^28^ However, G9A recruitment by GR, Runx2 and NF-E2/p45 leads to activation of target genes in a SET-domain-independent manner.^29^ The Air noncoding RNA may also recruit G9A to chromatin and epigenetically silences the transcription of target genes.^30^ It would be interesting to identify such recruiting factor(s).

Our study also indicates that CASP1 suppresses tumor cell invasion and migration in NSCLC cells. CASP1 is one of components of the inflammasome complex, which also includes PYCARD, NALP and caspase 5 (also known as ICH-3). They function in the innate immune system, and can activate inflammatory process^31^ and induce cell pyroptosis.^32^ Interestingly, several NALP proteins are also differentially expressed in NSCLC. How various components in the inflammasome affect tumorigenesis in NSCLC may need further investigation.

Overall, our results identify a novel mechanism by which G9A enhances tumor cell proliferation and invasion by silencing CASP1 expression, and suggests that G9A may serve as a therapeutic target in NSCLC.

## Materials and methods

### Ethics

All animal experiments were performed using male BALB/C nude mice (4–5 weeks old). The mice were purchased from the SLAC Laboratory Animal Center (Shanghai, China) and cared for in accordance with the National Institutes of Health Guide for the Care and Use of Laboratory Animals. All animal experimental protocols performed in this study were approved by the Institutional Animal Care and Use Committee at Tongji University.

### Cell culture

Human NSCLC cell lines, PC9 and A549, were grown in DMEM medium (HyClone, Logan, Utah, USA). Culture media contain 10% FBS (Carlsbad, CA, USA) supplemented with penicillin (100 U/ml) and streptomycin (100 mg/ml) (Life Technologies, Carlsbad, CA, USA). The cells were incubated at 37 °C in a humidified atmosphere of 5% CO_2_.

### siRNA or overexpression plasmid transfection

G9A siRNAs were synthesized by Ribobio Inc. (Guangzhou, China). Transfections were performed with Lipofectamine 2000 (11668019; Invitrogen, Carlsbad, CA, USA) according to the manufacturer's protocol. Total RNA or cell lysates were prepared 48 h after transfection and were used for real-time RT-PCR or western blotting (WB).

Sequences for siRNAs targeting G9A were as follows: #1: 5′-CCAUGCUGUCAACUACCAUdTdT-3′ (sense) and 5′-AUGGUAGUUGACAGCAUGGdTdT-3′ (antisense); and #2: 5′-GAACAUCGAUCGCAACAUCdTdT-3′ (sense) and 5′-GAUGUUGCGAUCGAUGUUCdTdT-3′ (antisense); and #3: 5′-GCUAUGAGGCUACUGAGUAdTdT-3′ (sense) and 5′-UACUCAGUAGCCUCAUAGCdTdT-3′ (antisense).

CASP1 siRNAs were synthesized by GenePharma Inc. (Shanghai, China). The sequences for siRNAs targeting CASP1 were as follows: #1: 5′-GGUGUGGUUUAAAGAUUCATT-3′ (sense) and 5′-UGAAUCUUUAAACCACACCTT-3′ (antisense); #2: 5′-GAAGACUCAUUGAACAUAUTT-3′ (sense) and 5′-AUAUGUUCAAUGAGUCUUCTT-3′ (antisense); and #3: 5′-CUCUCAAGGAGUACUUUCUTT-3′ (sense) and 5′-AGAAAGUACUCCUUGAGAGTT-3′ (antisense).

G9A and CASP1 overexpression plasmids and the control plasmid (GV141) were purchased from GeneChem Inc. (Shanghai, China). Plasmids were transfected into cells using Lipofectamine 2000 (11668019; Invitrogen) according to the manufacturer's protocol.

### Establishment of stable G9A knockdown cell lines

PC9 and A549 cells were infected with the lentiviral supernatant containing the lentiviral construct for G9A shRNA or the control shRNA (prepared by Target Inc., Shanghai, China). Puromycin was added to the cells for killing uninfected cells. Multiple single colonies were selected and expanded. Total RNA and cell lysates from these colonies were prepared and used for real-time RT-PCR or WB to validate the G9A knockdown effect in these cells. The detailed procedure was described as previously described.^33^

The oligonucleotide sequences used to make the shRNA against G9A were as follows: the forward oligo: GATCCGGCACACATTCCTGACCAGAGATGGTACCATCTCTGGTCAGGAATGTGTGTTTTTG and the reverse oligo: AATTCAAAAACACACATTCCTGACCAGAGATGGTACCATCTCTGGTCAGGAATGTGTGCCG. The two oligos were annealed and cloned into the lentiviral vector. The G9A-targeting sequence within these oligos is CACACATTCCTGACCAGAGAT.

### Cell proliferation assay

Cells were seeded into 96-well plates at a density of 1000 cells per well. For different treatment conditions described in the paper, each condition was replicated six times. At different time points, and cell proliferation assays were performed as previously described.^33^

### Cell invasion assay

Cell invasion assays were performed as previously described.^33^

### Cell migration assay

Cells were cultured in six-well plates until the cell density reached 80% confluence, and cell migration assays were performed as previously described.^33^ The assays were performed in triplicate.

### Colony formation assay

Cells were seeded into 10-cm dishes at a density of 1000 cells per dish and cultured in the presence of puromycin (0.125 *μ*g/ml) for 12 days, and colony formation assays were performed as previously described.^33^ The assays were performed in triplicate.

### Soft agar assay

Soft agar assays were performed as previously described.^33^

### Cell cycle analysis

Cells were centrifuged, washed twice with PBS and incubated with cold 70% ethanol at 4 °C overnight. Cells were mixed with PI-RNase staining buffer (BD Pharmingen, San Jose, CA, USA; #550825) according to the manufacturer's instructions. Stained cells were analyzed using a BD Accuri C6 Flow Cytometer (San Jose, CA, USA). Results were plotted using (FlowJo, LLC, Ashland, OR, USA).

### Plasmid cloning and luciferase reporter assay

The proximal promoter region (1.5 kb) of CASP1 gene was cloned into the pGL3 vector using the following PCR primers for genomic DNA amplification: the forward primer, 5′-CTAGGCTAGCACAGCAGCACTCCATTACTCAGTA-3′ and the reverse primer, 5′-GCCCTCGAGTAGCCTGCATCAGGTAGTGTATCC-3′. Luciferase reporter assays were carried out using a dual-luciferase reporter assay system (E1910; Promega), according to the manufacturer's instructions.

### Western blotting

WB was performed as previously described.^33^ GAPDH was used as a loading control. The following primary antibodies were used: G9A (CST, Danvers, MA, USA; #3306), H3K9me2 (CST; #4658), total histone H3 (CST; #4499), caspase 3 (CST; #9665) and PARP1 (CST; #9542), p-ERK (CST; #4370), ERK (Santa Cruz Biotechnology, Dallas, TX, USA; sc-94), GAPDH (Sigma-Aldrich, St. Louis, MO, USA; #G9545), *β*-Actin (CST; #4970). The following secondary antibodies were used: anti-rabbit IgG (CST; #7074S) and goat polyclonal anti-mouse IgG (Abcam, Cambridge, MA, USA; #ab136815).

### Apoptosis assay

Cells were washed twice in cold 1 × PBS twice. Then, 1 × 10^6^ cells per ml were resuspended in 1 × binding buffer, and 100 *μ*l of the cell suspension was mixed with 5 *μ*l FITC and 5 *μ*l PI using the FITC Annexin V apoptosis detection kit (BD 556547, San Jose, CA, USA), according to the manufacturer's instructions. Stained cells were analyzed using an Accuri C6 flow cytometer (BD Biosciences, San Jose, CA, USA).

### RNA isolation and real-time RT-PCR

Total RNA extraction from cells and real-time RT-PCR were performed as previously described.^33^

The PCR primers used were as follows: G9A forward, 5′-TACACCACTCATTGGGGATG-3′ G9A reverse, 5′-GGGAAGAGGGGAATGACTTT-3′ GAPDH forward, 5′-GAGTCAACGGATTTGGTCGT-3′ GAPDH reverse, 5′-TTGATTTTGGAGGGATCTCG-3′ CASP1 forward, 5′-GCTTTCTGCTCTTCCACACC-3′ and CASP1 reverse, 5′-TCCTCCACATCACAGGAACA-3′.

### Chromatin immunoprecipitation (ChIP)-qPCR

ChIP experiments were performed as previously described.^34^ The updated protocol can be found at http://research.hudsonalpha.org/Myers/. Anti-EHMT2/G9A antibody (Abcam; ab40542) and anti-Histone H3 dimethyl Lys9 (H3K9me2) antibody (Active motif, Carlsbad, CA, USA; #39239) were used. Purified DNA was resuspended in EB buffer for subsequent SYBR green-based real-time PCR. The following primers, which cover the proximal promoter region of CASP1 gene, were used: CASP1 #1 (+140)-Forward, GAACAGTGGTTCACATACTC; CASP1 #1 (+300)-Reverse, GCCTGCATCAGGTAGTGTAT; CASP1 #2 (−100)-Forward, GTGAGCCAAGGTCAAATAAC; CASP1 #2 (+100)-Reverse, GGGTAATGTATGTCCCTGTG; CASP1 #3 (−300)-Forward, CCTAGCAATTTGGGAGACCA; and CASP1 #3 (−100)-Reverse, TGCAGCCTCCACTTCCCAGG; CASP1 #4 (−1000)-Forward, GACACGTCTTACATGGGTGC; CASP1 #4 (−800)-Reverse, ATGAAGGCTGGTGATATGGT.

### *In vivo* xenograft assay

For the xenograft tumor growth assay, a total of 1 × 10^6^ cells suspended in 100 *μ*l PBS were injected subcutaneously into the right axillary region of 5-week-old nude mice. Tumor size was measured every 3–5 days using a digital caliper, and tumor volume (*v*) was calculated based on this formula: *v*=1/2(length × width^2^). After 5 weeks, xenograft tumors were isolated, photographed and fixed. The immunohistochemistry experiments were performed using the following primary antibody: Ki67 (ab15580; Abcam) by Wuhan Google Technology Co. Ltd. (http://servicebio.cn/, Wuhan, China). The H&E-stained slides were scanned using a Leica SCN400 (Wetzlar, Germany) and analyzed using SlidePath Gateway LAN software (Wetzlar, Germany).

### Small-molecule inhibitor

BIX-01294 was purchased from MedChemExpress (Monmouth Junction, NJ, USA, #HY-10587). PC9 and A549 cells were plated in six-well plates until the cell density reached 30–40% confluence, and the cells were then treated with different concentrations of BIX-01294 for various lengths of time.

### Statistical analysis

The RNA-Seq data of LUAD were obtained from the TCGA project (http://cancergenome.nih.gov) and the firebrowse (http://firebrowse.org). Differentially expressed genes were analyzed as previously described.^33^ Pearson's correlation was applied to analyze the correlation coefficient between two genes in the normal or cancer samples.

Comparison of pathway analysis was carried out in R (https://www.bioconductor.org/packages/devel/bioc/manuals/clusterProfiler/man/clusterProfiler.pdf) using gene expression correlation data downloaded from cBioportal (http://www.cbioportal.org/).

Gene expression and survival information for NSCLC patients were from the released database (2015 version) downloaded from http://kmplot.com/.^26^ Kaplan–Meier survival analysis was carried out using the statistics package for IBM SPSS version 22 (Armonk, NY, USA).

A comparison is made between the outcomes of the control condition and one of the treatment conditions unless stated otherwise. For comparisons between two groups, Student's *t*-test was used. For comparisons among multiple groups, one-way ANOVA was used. For all analyses, a *P*-value of <0.05 was considered statistically significant. *P<0.05, **P<0.01, ***P<0.001, ****P<0.0001.
