# Supplementary material for: G9A promotes tumor cell growth and invasion by silencing CASP1 in non-small-cell lung cancer cells
Source: Cell Death Dis. 2017 Apr 6;8(4):e2726–. doi: 10.1038/cddis.2017.65 (PMC5477595; doi:10.1038/cddis.2017.65)
Supplement: Supplementary Information [file cddis201765x1.docx]

**Supplemental figure legends:**

**Figure S1. Cell invasion, migration, and colony formation assays using siRNA targeting G9A**

(A) Validation of G9A knockdown by siRNA in PC9 and A549 cells transfected with either the control siRNA (si-Control) or G9A siRNA #2 (si-G9A); (B) Cell invasion assays of PC9 and A549 cells transfected with the control siRNA or G9A siRNA; (C, D) Cell migration assays of PC9 (C) and A549 (D) cells transfected with the control siRNA or G9A siRNA. (E) Colony formation assays of PC9 and A549 cells transfected with the control siRNA or G9A siRNA. (F) H&E staining images of xenograft tumors (upper panel), and IHC staining images of Ki67 in xenograft tumors (lower panel) derived from PC9 cells stably expressing G9A shRNA (PC9/sh-G9A) or the control shRNA (PC9/sh-Control).

**Figure S2.** **Correlation analysis of gene expression between G9A and CASP1 in different stages of LUAD.** (a) T1 stage: r = - 0.4197, P < 0.0001. (b) T2 stage: r = - 0.4480, P < 0.0001. (c) T3 stage: r = - 0.2552, P = 0.0834. (d) T4 stage: r = - 0.3107, P = 0.1954. (e) Normal tissues: r = - 0.1196, P = 0.3668. All the original data are from LUAD of the TCGA database. The number within the parenthesis represents the sample size.

**Figure S3. CASP1 expression is negatively or positively associated with distinctive cellular pathways in four different cancer types.** Four different cancer types include: lung adenocarcinoma (LUAD), breast cancer (BRCA), lung squamous carcinoma (LUSC), and colon adenocarcinoma (COAD). Numbers at the bottom of the table: #1 to #4 represent pathways enriched for genes with expression positively correlated with CASP1 expression in four cancer types (LUAD_Pos, BRCA_ Pos, COAD_ Pos, and LUSC_ Pos); #5 to #8 represent pathways enriched for genes with expression negatively correlated with CASP1 expression in four cancer types (LUAD_Neg, BRCA_Neg, COAD_Neg, and LUSC_Neg). The number within the parenthesis indicates the number of genes used for pathway analysis. Gene ratio is the number of genes in a pathway that meet cutoff criteria / total number of genes in the pathway, and adjusted p value is represented by the range of color from blue to red.

**Table S1. NSCLC patient survival analysis based on G9A and CASP1 expression.** (A) Patient survival data based on G9A and CASP1 expression. (B) Log rank test for the pair-wise comparison among four groups in patient survival analysis. Four groups are: NSCLC patients with high G9A and low CASP1 expression, high G9A and high CASP1 expression, low G9A and low CASP1 expression, and low G9A and high CASP1 expression. The data were downloaded from this website: <http://kmplot.com/>.
