# Supplementary figures and images for: G9A promotes tumor cell growth and invasion by silencing CASP1 in non-small-cell lung cancer cells
Source: Cell Death Dis. 2017 Apr 6;8(4):e2726–. doi: 10.1038/cddis.2017.65 (PMC5477595; doi:10.1038/cddis.2017.65)

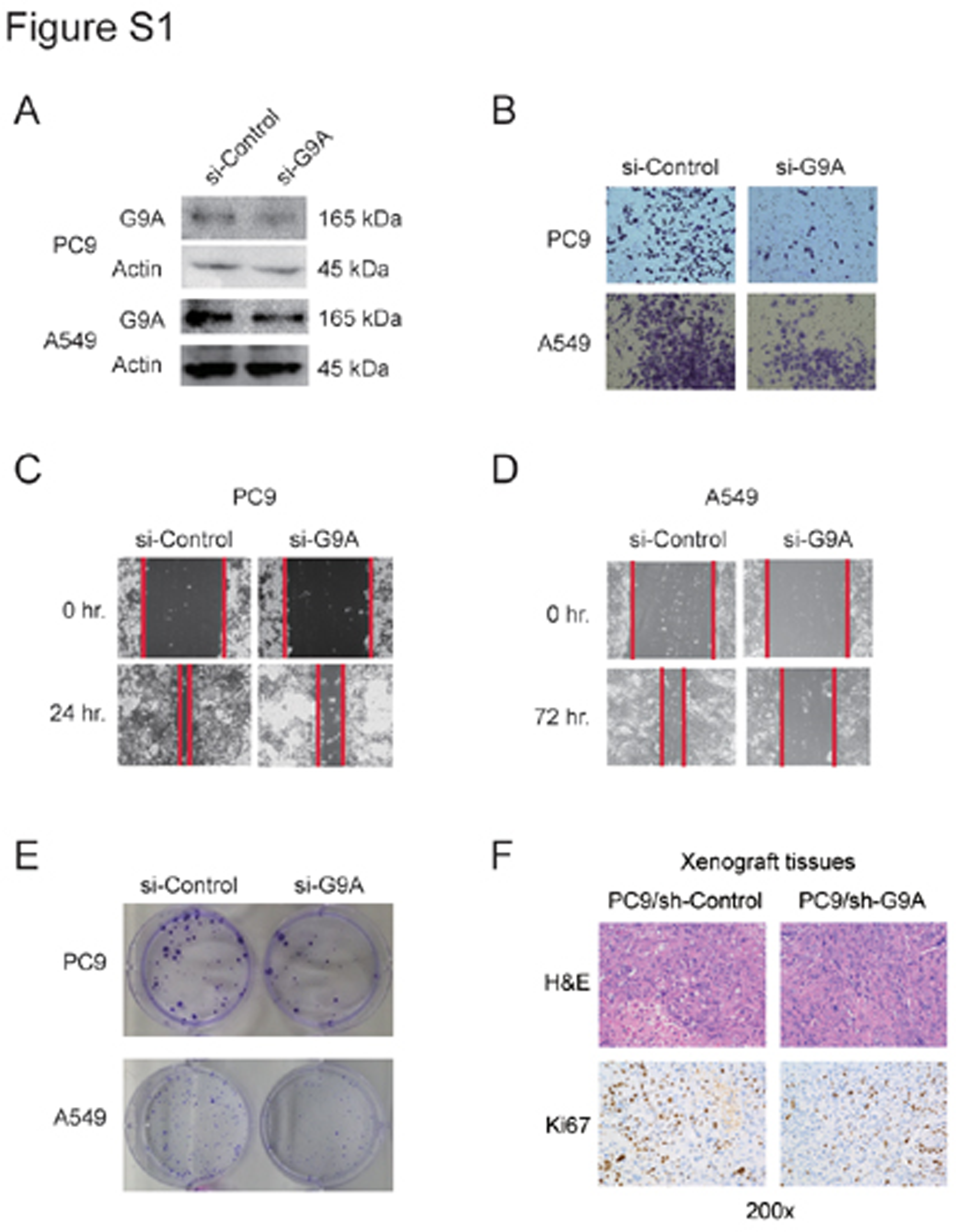

Supplement: Supplementary Figure 1 [file cddis201765x2.tif]

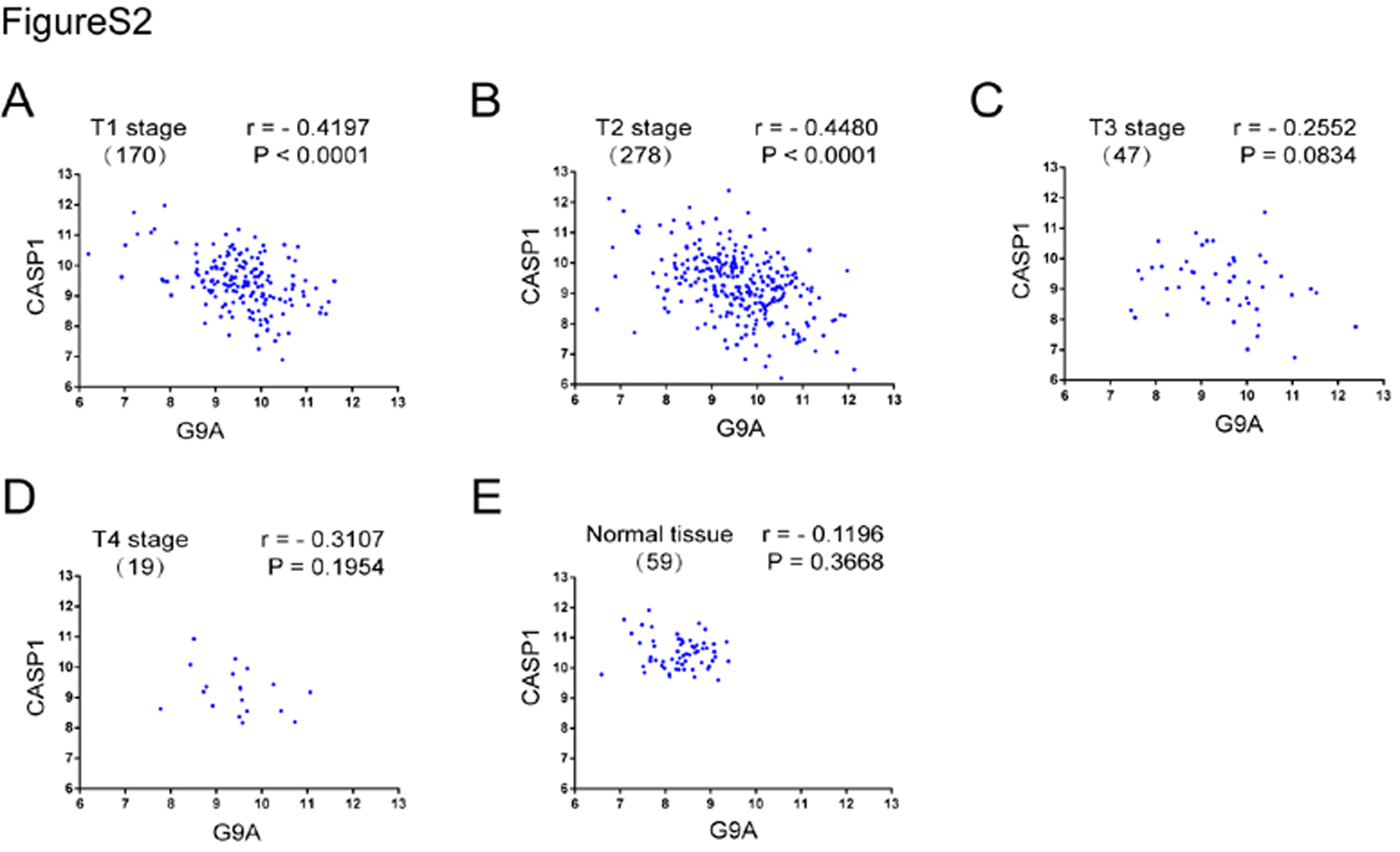

Supplement: Supplementary Figure 2 [file cddis201765x3.tif]

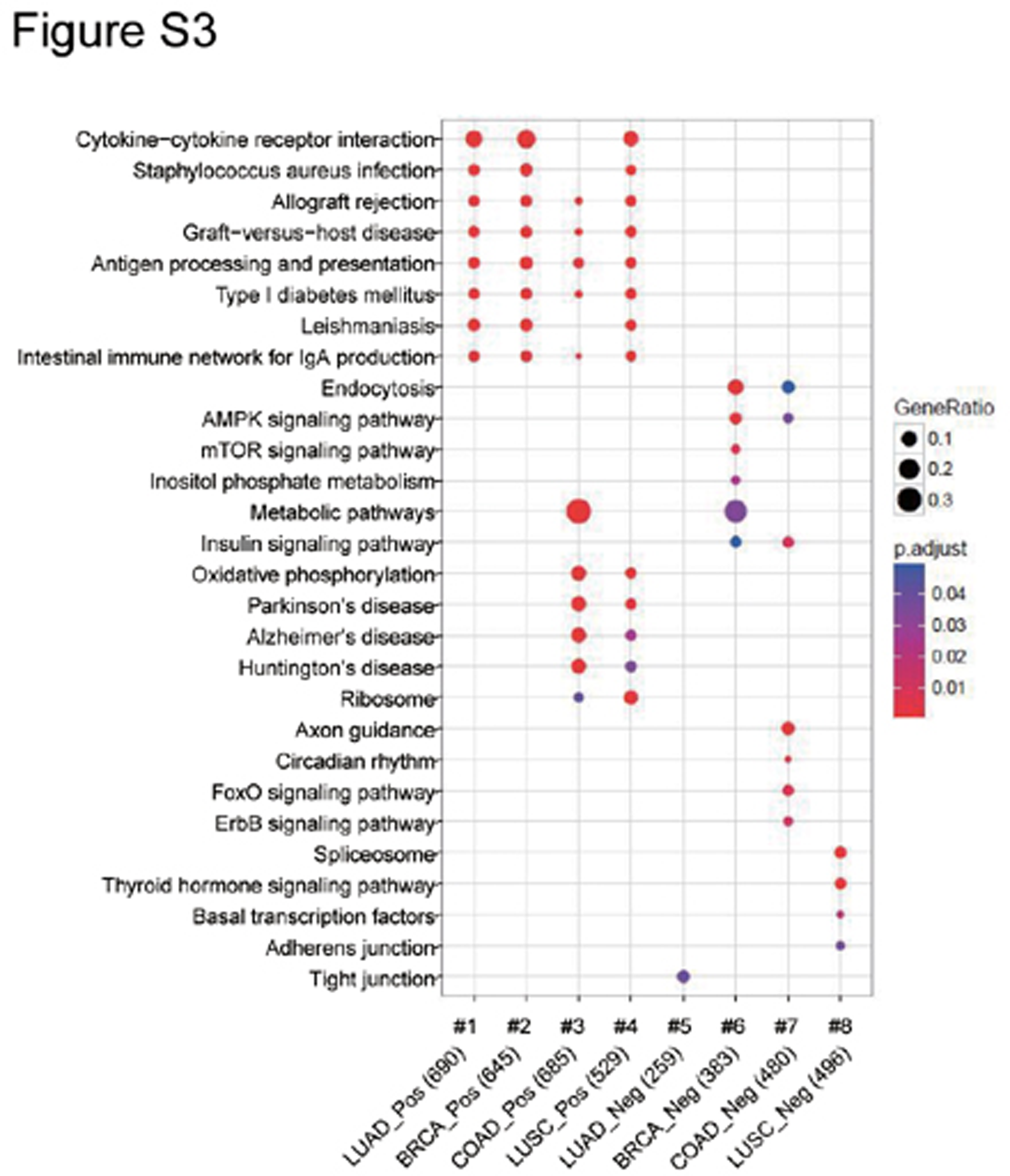

Supplement: Supplementary Figure 3 [file cddis201765x4.tif]
